# Sativex in resistant multiple sclerosis spasticity: Discontinuation study in a large population of Italian patients (SA.FE. study)

**DOI:** 10.1371/journal.pone.0180651

**Published:** 2017-08-01

**Authors:** Silvia Messina, Claudio Solaro, Isabella Righini, Roberto Bergamaschi, Simona Bonavita, Roberto Bruno Bossio, Vincenzo Brescia Morra, Gianfranco Costantino, Paola Cavalla, Diego Centonze, Giancarlo Comi, Salvatore Cottone, Maura Chiara Danni, Ada Francia, Alberto Gajofatto, Claudio Gasperini, Mauro Zaffaroni, Loredana Petrucci, Elisabetta Signoriello, Giorgia Teresa Maniscalco, Gabriella Spinicci, Manuela Matta, Massimiliano Mirabella, Graziella Pedà, Letizia Castelli, Marco Rovaris, Edoardo Sessa, Daniele Spitaleri, Damiano Paolicelli, Alfredo Granata, Mario Zappia, Francesco Patti, on behalf of the SA.FE. study group

**Affiliations:** 1 Department of Medical, Surgical Science and Advanced Technology "GF Ingrassia"–University of Catania, Catania, Italy; 2 Neurology Unit, Department Head And Neck, ASL3 Genova, Italy; 3 Department NEUROFARBA—University of Florence, Florence, Italy; 4 Department of Neurology—Neurology Institute C Mondino, Pavia, Italy; 5 I clinic Neurology—II University of Naples, Naples, Italy; 6 Neurology Operating Unit and Multiple Sclerosis Center—Provincial Health Authority of Cosenza, Cosenza, Italy; 7 Multiple Sclerosis Centre—University Federico II, Naples, Italy; 8 Demyelinating Diseases Centre—Foggia Hospital, Foggia, Italy; 9 A.O.U: Cittàdella Salute e dellaScienza di Torino, Torino, Italy; 10 Neuroscience Department—University Tor Vegata, Rome, Italy; 11 Unit of Neurology and of Neurorehabilitation, IRCCS Neuromed, Pozzilli (IS), Italy; 12 Department of Neurology–San Raffaele Hospital, Milan, Italy; 13 Neuroimmunology Unit—Villa Sofia-Cervello Hospital, Palermo, Italy; 14 Neurology Clinic—Ancona Hospital, Ancona, Italy; 15 Multiple Sclerosis Center, Dept. Neurol. Psich—Sapienza University, Rome, Italy; 16 Department of Neuroscience, Biomedicine and Movement Multiple Sclerosis Centre–University of Verona, Verona, Italy; 17 Neurology Division—San Camillo Hospital, Rome, Italy; 18 Multiple Sclerosis Centre—Sant'Antonio Abate Hospital, Gallarate, Italy; 19 Multiple Sclerosis Centre—University Hospital Pisa, Pisa, Italy; 20 Multiple Sclerosis Center—Second University of Naples, Naples, Italy; 21 Multiple Sclerosis Centre—Cardarelli Hospital, Naples, Italy; 22 Department of Medical Sciences—University of Cagliari, Cagliari, Italy; 23 Multiple Sclerosis Centre (CRESM)—San Luigi Gonzaga Hospital, Orbassano, Italy; 24 Multiple Sclerosis Centre—Cattolica University, Rome, Italy; 25 Multiple Sclerosis Centre—Vaio Hospital, Fidenza, Italy; 26 Multiple Sclerosis Centre—S. Andrea Hospital, Rome, Italy; 27 Multiple Sclerosis Centre—IRCCS Don Gnocchi Foundation, Milan, Italy; 28 Multiple Sclerosis Centre—IRCCS-Bonino Pulejo Centre, Messina, Italy; 29 Multiple Sclerosis Centre—San G. Moscati Hospital, Avellino, Italy; 30 Department of Basic Medical Sciences, Neuroscience and Sense Organs—University of Bari “Aldo Moro” Bari, Italy; 31 Department of Medical Sciences, Institute of Neurology—University “Magna Graecia”, Catanzaro, Italy; Heinrich-Heine-Universitat Dusseldorf, GERMANY

## Abstract

**Background:**

The approval of Sativex for the management of multiple sclerosis (MS) spasticity opened a new opportunity to many patients. In Italy, the healthcare payer can be fully reimbursed by the involved pharma company with the cost of treatment for patients not responding after a 4 week (28 days) trial period (Payment by Results, PbR), and 50% reimbursed with the cost of 6 weeks (42 days) treatment for other patients discontinuing (Cost Sharing, CS). The aim of our study was to describe the Sativex discontinuation profile from a large population of spasticity treated Italian MS patients.

**Methods:**

We collected data of patients from 30 MS centres across the country starting Sativex between January 2014 and February 2015. Data were collected from the mandatory *Italian Medicines Agency* (AIFA) web-registry. Predictors of treatment discontinuation were assessed using a multivariate Cox proportional regression analysis.

**Results:**

During the observation period 631 out of 1597 (39.5%) patients discontinued Sativex. The Kaplan-Meier estimates curve showed that 333 patients (20.8%) discontinued treatment at 4 weeks while 422 patients (26.4%) discontinued at 6 weeks. We found after adjusted modeling that a higher NRS score at T1 (adjHR 2.23, 95% 2.07–2.41, p<0.001) and a lower baseline NRS score (adjHR 0.51 95% CI 0.46–0.56, p<0.001) were predictive of treatment discontinuation.

**Conclusion:**

These data show that the first 6 weeks are useful in identifying those patients in which Sativex could be effective, thus avoiding the cost of longer term evaluation.

## Introduction

Spasticity is a common symptom in multiple sclerosis(MS) patients [1,2]. Spasms, pain, poor sleep quality, and urinary dysfunction are symptoms frequently associated with spasticity in MS [3]. The medications for generalized spasticity such as baclofen, tizanidine, dantrolene, and benzodiazepines, are not considered fully effective in reducing spasticity associated symptoms [4–6]. The approval of 9-delta-tetrahydocannabinol and cannabidiol (THC:CBD) oromucosal spray (Sativex),changed the management of spasticity and related symptoms in MS patients [7,8]. The efficacy of Sativex oromucosal spray as add-on therapy for symptoms improvement in MS patients with moderate to severe spasticity has been demonstrated in several clinical trials [9–11,7,12]. The largest pivotal phase III, enriched-design clinical trial included MS patients with moderate-severe spasticity, corresponding to a patient-reported 0–10 numerical rating scale (NRS) score ≥4, resistant to first line medications. After a first 4 weeks single-blind THC:CBD trial period, about 47% of the patients showed an initial response, and 2/3 of them kept on improving later on. In particular the patients after the first phase of the study showed a decreased of 3.01 points in the NRS score (from a baseline score of 6.91 to a score of 3.9). In the second phase of the study the patients in the active treatment group showed a further improvement of the NRS spasticity score (by 0.04 units from a baseline score of 3.87). The difference in mean NRS score between the placebo group and the active treatment group was statistically significant (0.84 points, p = 0.0002). About 50% of patients reported adverse events, being mild to moderate dizziness and fatigue the most common treatment-related adverse event [12]. In post-approval published observational studies patients tend to use lower doses compared with clinical trials (6–7 sprays/d vs. >8) and the incidence of adverse events was lower, with no evidence of addiction, abuse, misuse or memory impairment [13].

In Italy, the Italian Medicines Agency is adopting a reimbursement approach for new medications named Managed Entry Agreement (MEA). The Italian Medicines Agency agreement for Sativex considers both a payment by result (PbR) method and a cost sharing method (CS). The PbR consists of a complete reimbursement of all non-initial responder patients by the company (a 100% payback), whereas the CS consists of a 50% of reimbursement for patients on treatment for 6 weeks (GU Serie Generale n.100 del 30-4-2013—Suppl. Ordinario n. 33). According to the Italian Medicines Agency, the 4 weeks trial period “non-responders” have to discontinue treatment. For tracking these conditions, an e-Registry for all the patients starting Sativex was established in the Italian Medicines Agency website. Given the growing importance of data derived from real world observational studies, we decided to collect information about Sativex use in Italy, using the Italian medicine agency prospective e-registry designed to collect efficacy, tolerability and safety data. The Italian Medicines Agency e-registry, mandatory for all patients receiving Sativex in Italy, was used as a main prospective source database. It would bring a comprehensive picture of Sativex effect, minimizing biases. For further information, we also used complementarily the involved patients’ medical charts retrospective review. In a recent first publication derived from this database analysis, we found that Sativex was effective with no unexpected AEs, abuse and misuse. We found 70.5% of patients reached a ≥20% improvement (initial response, IR) and 28.2% reached a ≥30% improvement (clinically relevant response, CRR). The mean number of spray was 6.8 per day at T1. During the observation period a total of 631 patients (39.5%) discontinued treatment [14]. The main aim of this study was to further describe Sativex discontinuation and adverse events profile in this large population of Italian MS patients. In addition, we analyzed discontinuation time for lack of efficacy and adverse event reasons, considering the outcome-based risk and cost sharing Italian agreement time frames (4 weeks and 6 weeks).

## Material and methods

### Design and setting

All patients included in the study database were treated in accordance with the approved label and expected standards of good clinical practice. Complementary clinical and demographic parameters were acquired retrospectively from the patients’ medical records.

The study was approved by the Policlinico-Vittorio Emanuele (Catania, Italy) Ethics Committee (n° 37/2015/PO) and as required, by the Ethics Committee of the other participating centres. Given the observational design of the study, consent form was not required (according to the Italian law).

Patients were consecutively included in the e-registry at the start of Sativex treatment (baseline) and followed prospectively up to 6 months each, with data collection at baseline(T0), after 4 weeks (T1), after 12 weeks (3 months, T2) and after 24 weeks (6 months, T3) from baseline. We continued the observation up to 730 days (2 years since baseline) to register time to discontinuation according to our clinical practice. We considered the 4 weeks and 6 weeks to determine Sativex discontinuation time.

### Patient population

The AIFA registry establishes that patients are eligible for starting Sativex add-on treatment when fulfilling the following approved label related inclusion criteria: MS patients older than 18 years, with moderate to severe spasticity (0–10 Numerical Rating Scale (NRS) score ≥4) and not responding to common and ongoing antispastic drugs (used under their approved label). Other exclusion criteria were: severe cardiovascular diseases, past history of psychiatric diseases, use of street cannabis and/or other psychoactive drugs, pregnancy and MS spasticity NRS score <4.

The 30 MS participating centres collected all patients’data from the AIFA Sativex e-registry website. MS spasticity evolution was evaluated by the 0–10 NRS patient rated scale(0 = no, 10 = maximal spasticity)[15]. MS physical disability was evaluated using the expanding disability status scale(EDSS). Other parameters such as use of other antispastic drug, previous antispastic drugs and treatment discontinuation were collected. Furthermore, complementary demographical and clinical history data, tolerability, daily dose (n° of spray per day), overall clinical response to Sativex, discontinuation reason(s) and time to discontinuation were collected from patients’ medical charts. Data were manually entered in an *ad hoc* created database and were double entered into the database. Data cleaning was also performed before the data analysis. Tolerability was assessed collecting data, after prompted **question to the patient** during web registry data entry, about each adverse event (AE) and serious adverse event (SAE) occurring during the whole study period, in accordance with the pharmacovigilance regulations. **Dataset of all patients ‘records are available as supporting information (S1 File).**

### Statistical analysis

Data were analyzed using the STATA 11.0 software packages [16]. Data cleaning was performed before the data analysis considering both range and consistency checks.

Quantitative variables were described using means and standard deviations (SD). The difference between means and the difference between proportions were evaluated by the t-test and the Chi-square test respectively. A Shapiro-Wilk test was performed to assess the normal distribution of data. In case of not normal distribution appropriate non-parametric tests were performed. A Kaplan–Meier survival curve was plotted to provide estimates of treatment failure by time. The Log-rank test was used for the univariate analysis of categorical analysis whereas the Cox proportional hazard ratio was used for the univariate analysis of continuous variables. Predictors of treatment discontinuation were assessed using a multivariate Cox proportional regression analysis. We considered “treatment failure, yes/no” as the dependent variable and age, sex, disease duration, MS type, baseline EDSS, baseline NRS and NRS at T1 as independent variables. In the absence of discontinuation event, data were censored at the last observation day. Multivariate analysis was performed to investigate the independent effect of a risk or protective factor after adjustment for one or several other factors or to adjust for interactions between covariates, when significant. Parameters associated with the outcome at the univariate analysis with a threshold of p = 0.20–0.25 were included in the multivariate model. The multivariate modeling analysis was adjusted for age and sex. Interactions between covariates were examined in the multivariate models. The likelihood-ratio test was performed to compare the model with interactions and the model without interactions.

## Results

A total of 1615 patients with MS spasticity starting treatment with Sativex were recruited from 30 Italian MS large centers distributed geographically across the nation. Recruited patients started treatment between January 1^st^ 2014 and end of February 2015 (see Table 1 for demographics and clinical details). Out of the 1615 patients, 18 patients were excluded from the analyses because their baseline NRS score was not available, leading to a 1597 analyzable patients’ sample. A total of 704,009 exposed patients/days were analyzed, with a median follow-up time of 730 days (2 years) (range 2–730).

**Table 1:** Clinical and demographic data.

N	1597 patients
**Male**(%)	756 (47.3)
**Female**(%)	841 (52.6)
**Relapsing Remitting MS** (%)	311 (19.5)
**Secondary Progressive MS** (%)	1029 (64.4)
**Primary Progressive MS** (%)	255 (16)
**Age** (years, mean, range)	51 (21–84)
**Disease duration** (years, mean±SD)	17.5 ± 8.6
**Baseline EDSS** (median, range)	6.5(1.5–9.5)
**NRS score T0, Baseline**(n = 1597 pts,mean±SD)	7.5 ± 1.4
**NRS score T1, Month 1**(n = 1432 pts,mean±SD)	5.9 ± 1.6
**NRS T2, Month 3** (n = 889 pts,mean±SD)	5.1 ± 1.6
**NRS T3 Month 6** (n = 593 pts,mean±SD)	4.8 ± 1.7
**Dose,** puffs number **T1**(mean±SD)	6.8 ± 2.6
**Dose,** puffs number **T2** (mean±SD)	6.5 ± 2.6
**Dose,** puffs number **T3** (mean±SD)	6.3 ± 2.8

Legend: MS = Multiple Sclerosis, EDSS = Expanded Disability Status Scale, NRS = Numerical rating scale, T0 = baseline, T1 = after 1 month, T2 = after 3 months, T3 = after 6 months.

For 2 patients disease course was not available.

Out of the study population of 1597 patients, 631 (39.5%) discontinued therapy during the observation period. Out of them,396 patients (24.8% from the overall sample) did not reach the 20% NRS score improvement vs. baseline initial response (IR) threshold at T1. Out of the 631, a total of 374 patients do not reach T2 visit (discontinued after T1).

Reasons for discontinuation during the whole observation period were (multiple answer possible) lack of effectiveness (n = 371, 23.2%), adverse events (n = 260, 16.3%), non-adherence (n = 12, 0.8%), lost at follow-up (n = 7, 0.4%) patient’s choice (n = 5, 0.3%) or reasons not available (n = 32, 2%). For AE and SAE details see our previous paper [14].

The Kaplan-Meier survival curve showed that 333 patients (20.8%) discontinued treatment within 4 weeks while 422 (26.4%) discontinued within 6 weeks. Time to discontinuation was analyzed as hazard ratio (HR) to identify clinical and demographic characteristics predictive of a higher risk of treatment failure.

We found after adjusted modeling that the NRS score at T1 visit was predictive of treatment discontinuation (adjHR 2.23, 95% 2.07–2.41, p = 0.000). In other words, if the NRS score at T1 increases by one point, while the others variables are held constant, the hazard of Sativex discontinuation increases by two-fold. Additionally baseline NRS score was also associated to the outcome (adjHR 0.51 95% CI 0.46–0.56, p<0.001). In particular, as the baseline NRS score increases by one point, while the others variables are held constant, the hazard of Sativex discontinuation decreases by 49% (See Table 2).

**Table 2:** Multivariate analysis of predictors of discontinuation.

Discontinuation	adjOR	p	95% CI
NRS T0	0.51	<0.001	0.46–0.56
NRS T1	2.23	<0.001	2.07–2.41

Legend: NRS = Numerical Rating scale; T0 = baseline; T1 = after 1 month; adjOR = adjusted odds ratio.

**Fig 1:**
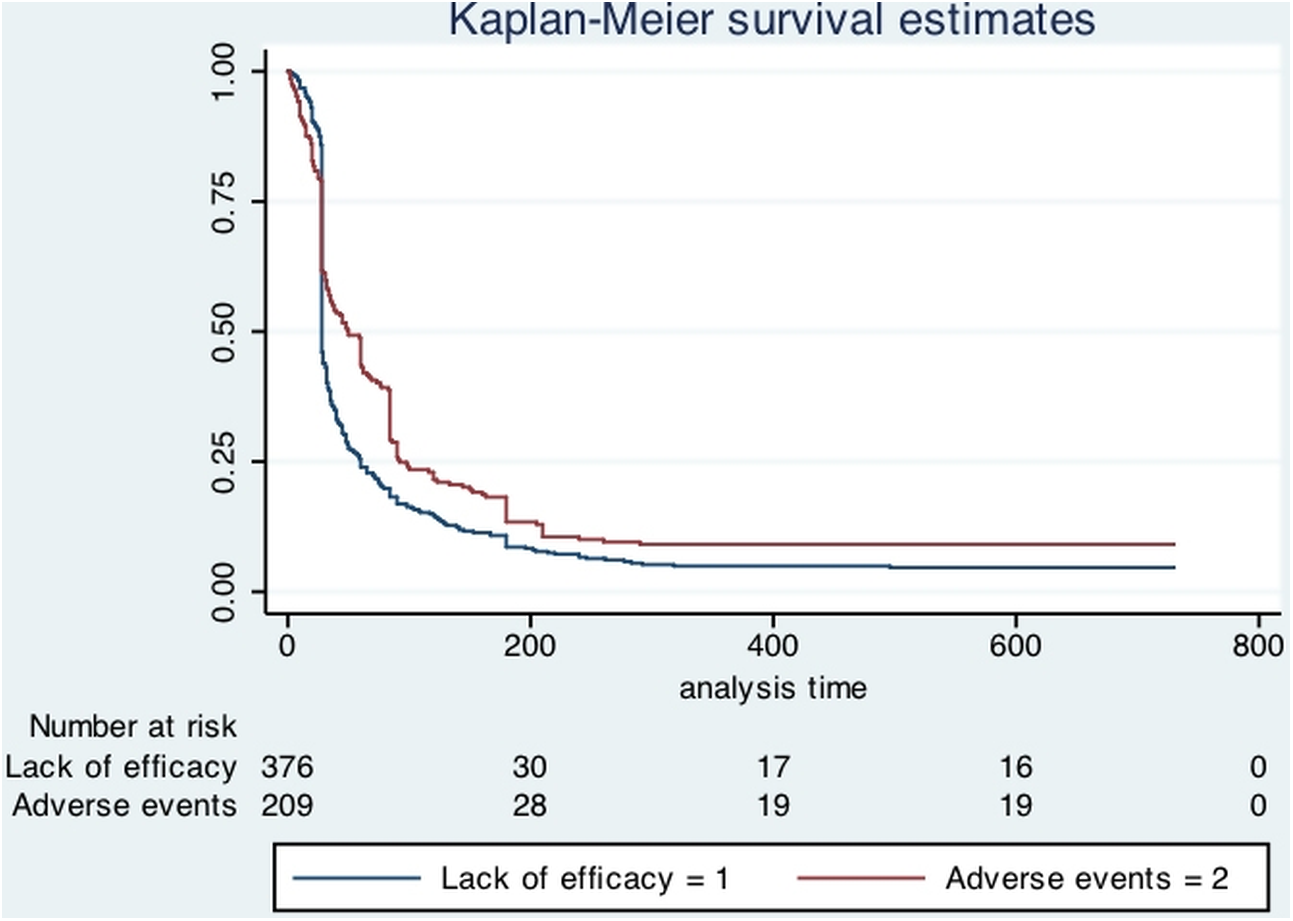
Kaplan-Meier survival estimates. Kaplan-Meier survival estimates showing the time to treatment failure for lack of efficacy and adverse events.

According to non-response or AE as reason for discontinuation we divided our cohort in two groups. When the patients discontinued for multiple reasons we considered “non-response” as the main reason of discontinuation. We found a total of 376 patients considered as non-responders and 209 patients who discontinued just for AE. A Kaplan-Meier survival analysis showed that 203 of the non-responder patients (53.9%) discontinued treatment after exactly 4 weeks (28 days) while 257 (68.3%) discontinued after 6 weeks (42 days). In the group of patients discontinuing just for AE, we found 80 patients (38.3%) discontinued after 4 weeks (28 days) while 97 (46.4%) within 6 weeks (42 days). A log-rank test showed a significant difference in survival function between these groups (p = 0.0016) (see Fig 1).

## Discussion

**Fig 2:**
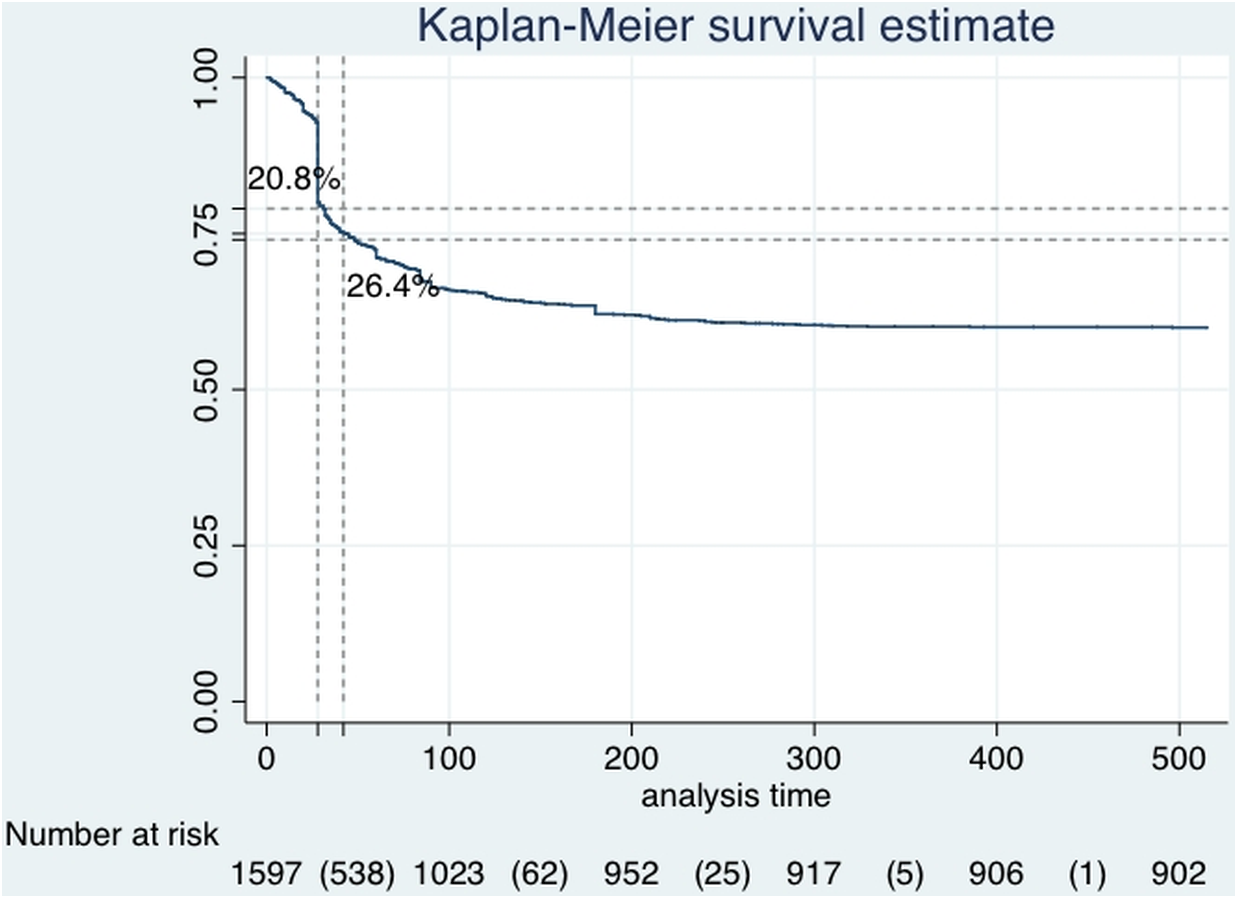
Kaplan-Meier survival estimates. Kaplan-Meier survival estimates showing the time to treatment failure for patients.

This study showed that 39.5% of patients discontinued Sativex during the whole observation period. In particular, almost 25% of those patients did not reach the 20% initial response reduction in their NRS score, considered the threshold for treatment effectiveness. Reasons for discontinuation during the whole observation period were lack of effectiveness for 23.2% of patients and adverse events for 16.3% of patients (see Fig 1). In the overall group of discontinuing patients the Kaplan-Meier survival curve showed that 20.8% (333) patients discontinued treatment after 4 weeks while 422 (26.4%) discontinued after 6 weeks (see Fig 2). These data suggest that the initial response (IR) threshold is appropriate for identifying those patients more prone to respond to Sativex effects, although survival estimates apparently may seem in contrast with discontinuation percentage (almost 25% did not reach IR). The Italian Medicines Agency web registry, always requiring the IR threshold before Sativex dispensation, could in part explain this assumption. It could be postulated that in the real life setting the NRS score evaluation was delayed beyond four weeks to better define the response to treatment. This could be in part demonstrated by the survival curve, showing 26.4% of patients discontinued after 6 weeks and by our previous study, showing a percentage of 9.3% of patients considered clinically partial responder by physicians at T1 [14].

These data showed that the 4 weeks trial is effective in identifying those patients where Sativex could be effective, thus limiting the economic burden of Sativex on the health system. To reduce costs and improve savings for the Italian health system, a response-based risk-sharing agreement between the Italian Medicines Agency and the company distributing Sativex, was implemented [17]. According to the agreement, the health system avoids the cost of early non-responders, being reimbursed if the outcome is not reached, saving resources. After the 4 weeks period it is seen a discontinuation rate of 20.4% of exposed patients, while after the 6 weeks period we see a 26.4% discontinuation rate. The pharmaceutical company will have to reimburse 100% of the patients failing at 4 weeks and 50% of all patients reaching 6 weeks (for both reasons). This means that the Italian Medicines Agency, related to our sample, will get fully reimbursed for the 20.4% patients not responding after 4 weeks and for 50% of those reaching 6 weeks, fulfilling the payment by results and cost sharing concepts [18]. The Italian Medicines Agency got reimbursed for about 66% of non-responder Sativex population within the first 6 weeks, suggesting the MEA represent a good tool for payers, avoiding the wasting of resources for patients not adequately responding. In particular considering the PbR reimbursement was 436.81 euros for 333 patients whereas the CS reimbursement was 218.40 euros for 89 patients, AIFA got a total reimbursement of 164,895.775 euros (145,457.73 + 19,438.045).

The multivariate analysis showed that the NRS score at T1 was predictive of treatment discontinuation (adjHR 2.23).

In other words, if the NRS at T1 increases by one point, the probability of Sativex discontinuation increases by two fold. This is in line with the initial responder definition, requiring a 20% improvement of NRS score at T1 compared to T0. Additionally, the baseline NRS score was also associated to the outcomes (adjHR 0.51). In particular, if the baseline NRS score increases by one point, the hazard of Sativex discontinuation decreases by 49%. Overall, higher NRS scores are related to a higher probability to be a responder and a lower probability to stop treatment. An increase of NRS score at T1 reflects the worsening of spasticity despite Sativex treatment, suggesting a higher probability of treatment discontinuation. This is in line with the results of our previous study showing higher NRS score is associated to a higher probability to be a responder [14]. However, as mentioned in our previous study, the observational design and the use of patient reported spasticity score, can in part affect our findings [19].

In conclusion, we observed that Sativex is a good option for a large number of MS patients complaining of moderate to severe resistant spasticity. The 4–6 weeks trial period is a reliable tool to identify those patients responding to treatment, limiting the economic burden of Sativex on the national health system, charging most of the effectiveness failure risks to the pharmaceutical company while adding a cost sharing side. Considering the increasing cost for the society of standard of care treatment of MS patients spasticity related disability and the negative impact on their quality of life, we may suggest Sativex should be considered as the add-on treatment of choice for MS patients failing to achieve enough relief with first-line classic antispastic oral options [20]. Further research is needed to verify if Sativex could be used as a treatment option in different spasticity conditions other than MS and for MS symptoms other than spasticity, as a first line option or as add-on.

## Supporting information

S1 File Dataset of all patients’ records used for the data analysis. (XLSX)
